# Supplementary material for: Efficient numerosity estimation under limited time
Source: PLoS Comput Biol. 2025 Mar 7;21(3):e1012790. doi: 10.1371/journal.pcbi.1012790 (PMC12021274; doi:10.1371/journal.pcbi.1012790)
Supplement: S1 Note — (PDF) [file pcbi.1012790.s001.pdf]

Supplementary Note 1. General specification and derivation of the logarithmic noisy encoding and Bayesian decoding model

The goal of this supplementary note is to introduce the noisy log-encoding Bayesian model that was elaborated in more detail elsewhere [1]. Additionally, we formalize the connection between the log-normal and power-law priors. The definitions and derivations specified here will serve as a basis to specify the information theoretical part of the model developed in Supplementary Note 2.

In this model, we assume that a stimulus of numerosity n generates an internal representation that is drawn from a distribution

$$r \sim N(\log n, \nu^2) \ , \tag{23}$$

where the noise parameter ν is independent of n. Here we assume that the prior distribution from which the numerosity value n is drawn is given by a log-normal distribution

$$\log n \sim N(\log \mu, \sigma^2) \ . \tag{24}$$

As stated in the main text, this distribution is qualitatively similar to the power-law distribution and also has many occurrences and applications in the statistics of human behavior. It is also generally present in various biological phenomena such as measures of length, area and weight of living organisms, and also present in neurophysiological observations such as distribution of firing rates across populations of neurons and intrinsic gain and synaptic weight in neural systems [2].

Based on these two assumptions (Eqs. 23 and 24), it follows that the distribution of $\log n$ conditional on the value of r will be a Gaussian distribution

$$\log n \mid r \sim N\left(\mu_{\text{post}}(r), \sigma^2_{\text{post}}\right) \ . \tag{25}$$

It follows that the conditional mean of $\log n$ is given by

$$\mu_{\text{post}}(r) = \mathrm{E}[\log n \mid r] = \mu + \beta \cdot (r - \mu) \ , \tag{26}$$

with the slope of this linear projection given by

$$\beta = \frac{\sigma^2}{\sigma^2 + \nu^2} \ . \tag{27}$$

And the conditional variance is given by

$$\sigma^2_{\text{post}} = \frac{\sigma^2 \nu^2}{\sigma^2 + \nu^2} \ . \tag{28}$$

Here we consider the hypothesis that the participant's numerosity estimate minimizes the MSE. Thus, the rule that is optimal under this objective is given where the estimate $\hat{n}$ is

defined by $\hat{n} = \mathrm{E}[n \mid r]$ for all r. It follows from the properties of the log-normal distribution that the posterior mean is given by

$$\mathrm{E}[n \mid r] = \exp\left(\mu_{\text{post}} + (1/2)\sigma^2_{\text{post}}\right) \ . \tag{29}$$

In this case, the Bayesian model predicts

$$\begin{aligned} \log \hat{n}(r) &= \log \mathrm{E}[n \mid r] = \mu_{\text{post}}(r) + (1/2)\sigma^2_{\text{post}} \\ &= \mu + \beta \cdot (r - \mu) + (1/2)\sigma^2_{\text{post}} \end{aligned} \tag{30}$$

Given that r is a random variable, it follows that $\hat{n}(r)$ is also random variable. Thus, $\log \hat{n}$ is normally distributed conditional on n

$$\log \hat{n} \sim N\left(\hat{\mu}(n), \hat{\sigma}^2\right) \ , \tag{31}$$

with the mean and variance of this conditional distribution given by

$$\begin{aligned} \hat{\mu}(n) &\equiv \mathrm{E}[\log \hat{n} \mid n] = \mu + \beta \cdot (\mathrm{E}[r \mid n] - \mu) + (1/2)\sigma^2_{\text{post}} \\ &= \mu + \beta \cdot (\log n - \mu) + (1/2)\sigma^2_{\text{post}} \\ \hat{\sigma}^2 &\equiv \mathrm{var}(\log \hat{n} \mid n) = \beta^2 \, \mathrm{var}(r \mid n) \\ &= \beta^2 \nu^2 = \frac{\sigma^4 \nu^2}{\left(\sigma^2 + \nu^2\right)^2} \ . \end{aligned} \tag{32}$$

It then follows from the properties of the log-normal distribution that the expected value and variance of the numerosity estimators are given by

$$\mathrm{E}[\hat{n} \mid n] = \exp\left(\hat{\mu}(n) + (1/2)\hat{\sigma}^2\right) \tag{33}$$

and

$$\mathrm{var}[\hat{n} \mid n] = \left[\exp\left(\hat{\sigma}^2\right) - 1\right] \cdot \exp\left(2\hat{\mu}(n) + \hat{\sigma}^2\right) \ . \tag{34}$$

Finally, we can use the these equations to compute the ratio between the standard deviation and the expected value of the posterior estimators, i.e., the coefficient of variation (Eq. 14 in main text)

$$\frac{\mathrm{SD}[\hat{n} \mid n]}{\mathrm{E}[\hat{n} \mid n]} = \sqrt{e^{\hat{\sigma}^2} - 1} > 0 \ . \tag{35}$$

This expression does not depend on n, and therefore the log-encoding Bayesian model delivers the property of *scalar variability* discussed in the main text.

Note that in these calculations, only the *normalized prior* $\tilde{p}(n) \equiv p(n)/p(1)$ matters, and in fact the Bayesian posteriors can be well-defined even in the case of an improper prior (for which $\tilde{p}(n)$ is well-defined, but there is no value for $p(1)$ such that the implied density function $p(n)$ will integrate to 1). All of the above calculations can be generalized to apply to any normalized prior of the form

$$\tilde{p}(n) \ = \ \exp(-\alpha(\log n) - \gamma(\log n)^2) \ , \tag{36}$$

for some parameters α, γ with $\gamma \geq 0$. If $\gamma > 0$, this corresponds to a log-normal prior, with $\mu = (1-\alpha)/(2\gamma)$, $\sigma^2 = 1/(2\gamma)$. If instead $\gamma = 0$ but $\alpha > 0$, this corresponds to an improper power-law prior, $p(n) \sim n^{-\alpha}$.

In this latter case, the posterior implied by an internal representation r is again log-normal, as in equation (25), but now with parameters

$$\mu_{\text{post}}(r) \;=\; r \,+\, (1-\alpha)\nu^2 \;, \qquad \sigma^2_{\text{post}} \;=\; \nu^2$$

as limiting cases of equations (26) and (28). It then follows that the Bayesian posterior mean estimate $\hat{n}(r)$ will be log-normally distributed conditional on the true value of n, as in equation (31), but with parameters

$$\hat{\mu}(n) \;=\; \log n \,+\, (\frac{3}{2} - \alpha)\nu^2 \;, \qquad \hat{\sigma}^2 \;=\; \nu^2$$

as limiting cases of the formulas given above. Hence the mean estimate will be given by

$$\mathrm{E}[\hat{n}\,|n] \;=\; An \;, \qquad \text{where } A \;\equiv\; \exp((2-\alpha)\nu^2) \;>\; 0 \;, \tag{37}$$

and the standard deviation of the estimates will again satisfy (Eq. 14), with the value of $\hat{\sigma}^2$ given above.

Thus even in the case of an improper prior of this kind, the optimal Bayesian estimate $\hat{n}(r)$ is well-defined, and we can derive the predicted distribution of $\hat{n}$ conditional on n, as a function of the model parameters. All priors in the family (Eq. 36) imply that the distribution of estimates should satisfy the property of scalar variability (Eq. 14). In the case of a log-normal prior ($\gamma > 0$), equation (33) implies that $\mathrm{E}[\hat{n}\,|n]$ will be a strictly concave function of n, greater than n for all n below some critical value, and smaller than n for all n above the critical value. In the limiting case of a power-law prior ($\gamma = 0$), instead, equation (37) implies that $\mathrm{E}[\hat{n}\,|n]$ should be proportional to n, with either overestimation for all n (if $\alpha < 2$) or underestimation for all n (if $\alpha > 2$). In the special case of a power law with $\alpha = 2$, the model implies that the optimal Bayesian estimate should be unbiased for all n.

References

[1] Mel Win Khaw, Ziang Li, and Michael Woodford. “Cognitive Imprecision and Small-Stakes Risk Aversion”. In: *The Review of Economic Studies* (Aug. 2020). ISSN: 0034-6527. DOI: 10.1093/restud/rdaa044. URL: https://academic.oup.com/restud/advance-article/doi/10.1093/restud/rdaa044/5880002.

[2] Eckhard Limpert, Werner A. Stahel, and Markus Abbt. “Log-normal distributions across the sciences: keys and clues”. In: *BioScience* 51.5 (2001), pp. 341–352. DOI: 10.1641/0006-3568(2001)051[0341:LNDATS]2.0.CO;2.
