# Supplementary material for: Efficient numerosity estimation under limited time
Source: PLoS Comput Biol. 2025 Mar 7;21(3):e1012790. doi: 10.1371/journal.pcbi.1012790 (PMC12021274; doi:10.1371/journal.pcbi.1012790)
Supplement: S2 Note — (PDF) [file pcbi.1012790.s002.pdf]

Supplementary Note 2. Logarithmic noisy encoding and Bayesian decoding under limited informational capacity and temporal sensory exposure

In this supplementary note we show that it is possible to formulate an efficient coding model of numerosity estimation as developed in Supplementary Note 1, but in which encoding precision depends on stimulus viewing time t.

Instead of assuming, as in Supplementary Note 1, that a stimulus of numerosity n results in an internal representation r that is a single draw from a probability distribution that depends on n, we suppose now that the internal representation r instead consists of the sample path of a Brownian motion z_s over a time interval $0 \leq s \leq \tau$, starting from an initial value $z_0 = 0$. The drift m of the Brownian motion is assumed to depend on n, while its instantaneous variance ω^2 is independent of n; the length of time τ for which the Brownian motion evolves is also independent of n, but depends on the viewing time t. In assuming sensory evidence given by a Brownian motion with a drift that depends on the stimulus, we follow a long modeling tradition that includes the popular drift-diffusion model [2]. Models of this kind have been used since Taylor, Lindsey and Forbes [3] to account quantitatively for the way in which the accuracy of perceptual judgments is affected by manipulations of viewing time.

More specifically, we assume that m is an affine transformation of the logarithm of n,

$$m \;=\; \xi + \psi \log n \;, \tag{38}$$

where the parameters ξ and ψ may depend of the statistics of a particular environment. We suppose that the choice of these coefficients is subject to a "power constraint" which requires the average value of m^2 to be within some finite bound [1]

$$\mathrm{E}[m^2] \;\leq\; \Omega^2 \;<\; \infty \;. \tag{39}$$

This bound on the amount of variation in the drift limits the precision with which different stimuli can be discriminated, for any given τ. The value of τ is assumed to grow linearly with the viewing time, up to some time bound t_{max},

$$\tau \;=\; \min(t,\, t_{max}) \;, \tag{40}$$

representing a constraint on the amount of evidence that can be acquired by the brain. The latter bound constrains the degree to which precision can be increased by further increases in viewing time, just as in the TIM model.

For any fixed value of τ, the final position z_τ of the Brownian motion at time τ is a sufficient statistic for the information contained in the sample path about the value of n. Hence Bayesian decoding of the information contained in the sample path will yield the same

[1] The rationale behind the choice of the squared m is the following: The signals can take any value over the real line. If we assume that the status quo of no-message (or no energy being spent) is 0, then any deviations from 0 that lead to decodable information should be considered including negative values leading to energy expenditure.

result as if the internal representation is assumed simply to be the scalar random variable z_τ, with distribution

$$z_\tau \sim N(m(n)\tau, \omega^2\tau) . \tag{41}$$

Alternatively, we may suppose that the internal representation of n is given by the scalar random variable $r \equiv z_\tau/\tau$, which contains the same information as the variable z_τ. Under this representation of the sensory evidence, r is a draw from a distribution

$$r \sim N(m(n), \omega^2/\tau) . \tag{42}$$

Equation (42) effectively states that r is the output of a *Gaussian channel* with input m [1]; hence the problem of optimally choosing the function $m(n)$ is equivalent to an *optimal encoding* problem for a Gaussian channel. The capacity C of such a channel is a quantitative upper bound on the amount of information that can be transmitted regardless of the encoding rule, which is equal to

$$C = \frac{1}{2}\log\left(1 + \frac{\Omega^2 t}{\omega^2}\right) , \tag{43}$$

an increasing function of Ω/ω as well as of t. Here we suppose that the goal is to design a system that minimizes the mean squared error of the estimate $\hat{n}$ when n is drawn from a log-normal prior distribution (Eq. 24).

$$\log n \sim N(\log\mu, \sigma^2) . \tag{44}$$

Note that the estimate $\hat{n}$ depends on r_n. We re-express r_n as a function of the transformed variable $\tilde{r}_n \equiv (r_n - \xi)/\psi$, thus we can equivalently treat $\tilde{r}_n$ as the internal representation, and it follows that $\tilde{r}_n \sim N(\log n, \omega^2/(t\psi^2))$. Following the definitions provided in Supplementary Note 1, it follows that we have a noisy log-encoding with variance $\nu^2 = \omega^2/(t\psi^2)$. It then follows that the MSE in the case of any encoding rule is given by Eq. 30, and the MSE associated with this rule will be given by

$$\text{MSE} = \exp\left(2\mu + 2\sigma^2\right) \cdot \left[1 - \exp\left(-(1-\beta)\sigma^2\right)\right] . \tag{45}$$

Recall that β is a decreasing function of ν (Eq. 27), and therefore in order to make the MSE as small as possible it is desirible to make ν as small as possible. Given that $\nu^2 = \omega^2/(t\psi^2)$ is follows that we would like to make ψ as large as possible, consistent with the power constraint in Eq. 39. Thus, for the case of the log-normal prior the power constraint becomes

$$(\xi + \psi\mu)^2 + \psi^2\sigma^2 \leq \Omega^2 . \tag{46}$$

The maximum value of ψ consistent with this constraint is achieved when

$$\xi = -\psi\mu , \quad \psi = \frac{\Omega}{\sigma} . \tag{47}$$

In this case the encoding noise is given by

$$\nu = \frac{\omega}{\Omega\sqrt{t}}\sigma . \tag{48}$$

Defining $R \equiv \Omega/\omega$, we can define the encoding noise of numerosity estimation

$$\nu(t) = 1/G \ , \tag{49}$$

where

$$G = \min(R\sqrt{t}/\sigma, B) \ , \tag{50}$$

with B a maximum biologically allowed bound on sensory precision (similar to the assumption of the TIM model).

The precision of numerosity encoding is given by $\nu(t) = 1/G$, where $G = \min(R\sqrt{t}/\sigma, B)$ and B a maximum biologically allowed bound on sensory precision related to t_{max} ($B = R\sqrt{t_{max}}/\sigma$).

References

[1] Thomas M Cover. *Elements of information theory*. John Wiley \& Sons, 1999. DOI: 10.1002/047174882X.

[2] Roger Ratcliff. "A theory of memory retrieval." In: *Psychological review* 85.2 (1978), p. 59. DOI: 10.1037/0033-295X.85.2.59.

[3] M. M. Taylor, P. H. Lindsay, and S. M. Forbes. "Quantification of shared capacity processing in auditory and visual discrimination". In: *Acta Psychologica* 27 (1967), pp. 223–229. ISSN: 00016918. DOI: 10.1016/0001-6918(67)99000-2. URL: /record/1967-09813-001.
