# Supplementary material for: Efficient numerosity estimation under limited time
Source: PLoS Comput Biol. 2025 Mar 7;21(3):e1012790. doi: 10.1371/journal.pcbi.1012790 (PMC12021274; doi:10.1371/journal.pcbi.1012790)
Supplement: S3 Note — (PDF) [file pcbi.1012790.s003.pdf]

Supplementary Note 3. Recapitulation of the Gaussian channel capacity derivation

For convenience to the reader, the goal of this supplementary note is to recapitulate the derivation of the Gaussian channel capacity presented in Cover and Thomas [1] based on the notation used in our work, thus clarifying the connection to the solution of our SEB model under capacity constraints derived in Supplementary Note 2.

Suppose that Y is the output of a channel with input $X + Z$, where X is the signal and Z the noise. We assume the noise is drawn from a Gaussian distribution with variance ω^2/t and mean 0.

The goal is to find the maximum achievable channel capacity C by maximizing the mutual information $I(X;Y)$ for a given power constraint Ω^2

$$C = \max_{f(x):EX^2 \leq \Omega^2} I(X;Y) \tag{51}$$

It can be shown that

$$\begin{aligned} I(X;Y) &= h(Y) - h(Y \mid X) \\ &= h(Y) - h(X + Z \mid X) \\ &= h(Y) - h(Z \mid X) \\ &= h(Y) - h(Z) \ , \end{aligned} \tag{52}$$

where in general $h(X)$ is defined as the entropy of the channel X. Here, we will use two results. First, the entropy of the input of a Gaussian channel, for a given noise Z with variance ω^2, is given by

$$h(Z) = \frac{1}{2} \log 2\pi e \omega^2/t \ . \tag{53}$$

Second,

$$EY^2 = E(X+Z)^2 = EX^2 + 2EXEZ + EZ^2 = \Omega^2 + \omega^2/t \ , \tag{54}$$

given that X and Z are independent and $EZ = 0$. This means that the entropy of Y is bounded by

$$h(Y) \leq \frac{1}{2} \log 2\pi e(\Omega^2 + \omega^2/t) \ . \tag{55}$$

Thus, replacing Eqs. 53 and 55 in Eq. 52 gives

$$\begin{aligned} I(X;Y) &= h(Y) - h(Z) \\ &\leq \frac{1}{2} \log 2\pi e(\Omega^2 + \omega^2/t) - \frac{1}{2} \log 2\pi e \omega^2/t \\ &= \frac{1}{2} \log \left(1 + \frac{\Omega^2 t}{\omega^2}\right) \ . \end{aligned} \tag{56}$$

References

[1] Thomas M Cover. *Elements of information theory*. John Wiley \& Sons, 1999. DOI: 10.1002/047174882X.
