# Supplementary material for: Efficient numerosity estimation under limited time
Source: PLoS Comput Biol. 2025 Mar 7;21(3):e1012790. doi: 10.1371/journal.pcbi.1012790 (PMC12021274; doi:10.1371/journal.pcbi.1012790)
Supplement: S4 Note — (PDF) [file pcbi.1012790.s004.pdf]

Supplementary Note 4. Influence of the prior on the crossover point

The crossover point corresponds to the numerosity at which participants switch from over-estimation to underestimation. Here, we use the analytical solutions of the SEB model to show how the crossover point depends on elements of the prior distribution and the capacity constraints. More specifically, we can find the numerosity n^* at which the crossover will occur. In other words we can find the numerosity at which the following equality holds

$$n - E[\hat{n}|n] = 0 \ , \tag{57}$$

or alternatively

$$log(n) - log(E[\hat{n}|n]) = 0 \ . \tag{58}$$

Using the solutions in Eqs. 25, 26 and 31 it is possible to show that

$$n^* = \mu + \frac{1}{2(1-\beta(\sigma,\nu(t)))}[\sigma^2_{\text{post}}(\sigma,\nu(t)) + \sigma^2(\sigma,\nu(t))] \ , \tag{59}$$

where recall that μ is the prior location, σ is the prior spread, and $\nu(t)$ is the endogenized encoding noise function for a given time t (and also for a given encoding capacity). Note that none of the elements of expression in the second term in the right-hand side of the equality depend on the prior location μ, which means that changes in μ without changes prior spread or noisy coding elements will induce shifts in the direction of the prior location. This also means, and while not obvious from this expression, that positive changes in σ will induce a positive shift of the crossover point.
