# Supplementary material for: Efficient numerosity estimation under limited time
Source: PLoS Comput Biol. 2025 Mar 7;21(3):e1012790. doi: 10.1371/journal.pcbi.1012790 (PMC12021274; doi:10.1371/journal.pcbi.1012790)
Supplement: S5 Note — (PDF) [file pcbi.1012790.s005.pdf]

Supplementary Note 5. Comparison between the family TIM and SEB models

In this supplementary note, we compare the kind of information theoretical model that does not incorporate Bayesian inference (the TIM model defined in the main text), and the general family of Bayesian encoding-decoding models. This illustrative comparison is not directly applicable to our numerosity estimations, but can be solved analytically and is instructive. Nevertheless, we provide numerical simulations that are applicable to numerosity estimation in the main text which confirm the main predictions presented in this note (see Fig 2 in main text).

Illustrative comparison between the family of TIM and SEB models

In this illustrative example, the two models can be solved analytically, thus allowing to highlight the commonalities and differences between both models in an intuitive manner.

The TIM model proposes a method to infer how the distribution of estimates $\hat{n}$ should vary depending on the true stimulus magnitude n. The goal is to find the response distribution $p(\hat{n}|n)$ that minimizes the mean squared error (MSE)

$$\text{MSE} \equiv \int_N p(\hat{n} \mid n)(\hat{n} - n)^2 d\hat{n} \tag{60}$$

subject to the constraint that (Eq. 18 in main text)

$$D_{KL}\left(p_n \| q\right) \leq C(t) \tag{61}$$

where p_n is the distribution of possible responses conditional on n, q is the "prior" distribution, $D_{KL}\left(p \| q\right)$ is the Kullback-Leibler divergence, and $C(t)$ is a positive bound that depends on the amount of time t for which the stimulus is presented.

The TIM model can be developed further by specifying that q is given by the prior distribution from which n is expected to be drawn, and that $C(t)$ increases linearly with time, i.e., that $C(t) = c \cdot t$ for some $c > 0$, up to some finite bound B. Assuming that the prior is known, the model thus has only a single free parameter (the value of c) to predict the distribution of responses, as a function of both n and t, for all values of t below some upper bound.

We compare the predictions of this kind of model to the alternative Bayesian model, according to which (i) estimates are based on a noisy internal representation r of the stimulus magnitude n, which consists of a sequence of independent draws of a signal, the distribution of which depends on n, and with the number of draws in the sequence growing with t; and (ii) given the noisy internal representation, the participant's estimate is given by $\hat{n}(r) = \text{E}[n \mid r]$: Note that the computation of this last conditional expectation must be relative to a particular prior distribution from which n is expected to be drawn. Given the distribution of possible samples r for each n and t, we can use the assumed response rule to derive a predicted distribution of responses $\hat{n}$ for any specification of (n, t).

We make the Bayesian model example more specific by assuming that r is the cumulative value at time t of a Brownian motion that starts from the initial value $r = 0$, with a drift

$m(r)$ that depends on the stimulus and an instantaneous variance ω^2 that is independent of the stimulus. If we further assume that $m(n) = \mu \cdot n$ for some $\mu > 0$; then the model's predictions depend only on a single parameter, the value of $\gamma \equiv \mu/\omega$; again assuming that the prior is known. We thus have two one-parameter models, each of which makes precise predictions for the distribution $p_t(\hat{n} \mid n)$ for any n and t. Thus, in each model, the single free parameter determines how rapidly the precision of estimates should improve with increasing viewing time.

In this example, we suppose that the prior distribution for n is Gaussian, and let it be given by $N(0, \sigma^2)$. Here, we economize in notation by assuming that the prior mean is zero; the formulas that follow hold regardless of this, but n should be understood as the stimulus magnitude relative to the prior mean, and $\hat{n}$ as the response relative to the prior mean.

In the case of the Bayesian model, the information contained in the noisy internal representation is equivalent to that for a model in which the available information is a noisy measurement, $r \sim N(n, (\gamma^2 t)^{-1})$, the precision of which grows linearly with t. The optimal Bayesian estimate is then $\hat{n}(r) = \phi_t \cdot r$, where

$$\phi_t \equiv \frac{\sigma^2}{\sigma^2 + (\gamma^2 t)^{-1}} \ . \tag{62}$$

From this, it follows that the conditional distribution of responses for any time t will be given by

$$\hat{n} \mid n, t \sim N\left(\phi_t n, \phi_t \left(1 - \phi_t\right) \sigma^2\right) \ . \tag{63}$$

For the TIM model instead: the first order conditions for minimization of Eq. 19 subject to Eq. 18 require that

$$(\hat{n} - n)^2 + \theta_{n,t} \ln \frac{p(\hat{n} \mid n)}{q(\hat{n})} = k_{n,t} \ . \tag{64}$$

for all $\hat{n}$, where $\theta_{n,t}$ is the Lagrange multiplier associated with the capacity constraint given in Eq. 18 for given choices of n and t, and $k_{n,t}$ is a constant of integration. This equation can be solved for $p(\hat{n}|n)$ for each $\hat{n}$, given values for $k_{n,t}$ and $\theta_{n,t}$. We choose $k_{n,t}$ so as to imply a PDF $p(\hat{n}|n)$ that integrates to 1. We see that the resulting distribution for $\hat{n}$ is Gaussian

$$\hat{n} \mid n, t \sim N\left(\phi_{n,t}, \left(1 - \phi_{n,t}\right) \sigma^2\right) \ , \tag{65}$$

where the bias coefficient $\phi_{n,t}$ corresponds to

$$\phi_{n,t} = \frac{2\sigma^2}{\theta_{n,t} + 2\sigma^2} \ . \tag{66}$$

The value of $\theta_{n,t}$ is chosen so as to imply that the constraint given in Eq. 18 holds with equality. Computing the KL divergence, we see that this holds if and only if

$$\Gamma\left(\phi_{n,t}\right) + \phi_{n,t}^2 \frac{n^2}{\sigma^2} = 2C(t) \ , \tag{67}$$

where

$$\Gamma(\phi) \equiv - \ln(1 - \phi) - \phi \tag{68}$$

for any $0 < \phi < 1$.

For any n, we note that $\Gamma(\phi)$ is a continuous, monotonically increasing function of ϕ, approaching 0 as $\phi \to 0$ and becoming unboundedly large as $\phi \to 1$. Hence, for any n and any $C(t) > 0$, equation 67 has a unique solution satisfying $0 < \phi_{n,t} < 1$. We further observe that for a fixed value of n, increasing $C(t)$ increases the value of $\phi_{n,t}$; and for a fixed $C(t)$, increasing the value of $|n|$ increases the value of $\phi_{n,t}$.

Based on these analytical solutions, these results reveal some important similarities between the predictions of the TIM and the Bayesian models: for large enough t and allowing $C(t)$ to grow as function of t, then both models imply

1. $\mathrm{E}[\hat{n} \mid n] \to n$, and

2. $\mathrm{var}[\hat{n} \mid n] \to 0$.

Nonetheless, there are also several notable differences in the predictions of the two models. Here we provide a detailed explanation of these differences which were already mentioned in the main text:

1. *The quantitative dependence of estimation bias on viewing time.* While both models predict that $\phi_{n,t}$ should increase from 0 (for $t = 0$) to 1 (as $t \to \infty$), they do not imply the same rate of increase in $\phi_{n,t}$ as t increases. The Bayesian model implies that

$$\frac{\phi_t}{1-\phi_t} = \sigma^2\gamma^2 t \ , \tag{69}$$

   for any n. Hence for small t, $\phi_t \sim t$, while for large t, $(1-\phi)^{-1} \sim t$. Instead, if in the TIM model we assume that $C(t) = c \cdot t$ for all t, then for any $n \neq 0$, one can show that the solution to Eq. 67 satisfies $\phi_{n,t} \sim t^{1/2}$ for small t, while $(1-\phi)^{-1} \sim e^{2ct}$ for large t. Thus regardless of the parameters γ and c for the two models, we see that the TIM model implies faster growth of $\phi_{n,t}$ as t increases, both for sufficiently small values of t and for sufficiently large values of t.

2. *The relationship between estimation bias and the variability of estimates.* Again fixing some single value of $n \neq 0$, and considering the implied distribution of estimates for different viewing times, we see that the two models do not imply that $\mathrm{var}[\hat{n}|n,t]$ covaries with the bias in the same way. The TIM model implies that the variance falls monotonically with increases in $\phi_{n,t}$ (and hence that the variance falls monotonically with time, for any n). The Bayesian model instead implies that increases in ϕ_t first increase variance (while ϕ remains below 1/2), and then reduce variance again (once $\phi_t > 1/2$). The difference in predictions is especially stark in the case of small viewing time. As $t \to 0$, the Bayesian model's variance falls to zero (estimates are equal to the expected value of the prior), while the TIM model implies that this is the case in which estimates should be most variable (estimates are simply samples from the prior distribution, regardless of the value of n).

There are other differences between the two models that we do not highlight here as they are not strictly relevant to the discussion of this article.

Taken together, we developed an example in which analytical analyses allowed us to examine commonalities and differences between the two models. While the exact predictions of these differences do not hold for the specific application of the numerosity estimation models developed for the TIM model and the noisy log-encoding Bayesian model (see Supplementary Notes 1 and 2), these general differences make the two numerosity models identifiable, and thus generate different qualitative predictions. In particular, the two differences highlighted above cause the TIM model not to provide a general account of the scalar variability principle. That is, the ratio between variability and expected value estimations grows more rapidly in the TIM model relative to the log-encoding Bayesian model.
