# Supplementary material for: Efficient numerosity estimation under limited time
Source: PLoS Comput Biol. 2025 Mar 7;21(3):e1012790. doi: 10.1371/journal.pcbi.1012790 (PMC12021274; doi:10.1371/journal.pcbi.1012790)
Supplement: S1 Fig — (PDF) [file pcbi.1012790.s006.pdf]

Supplementary Figure 1

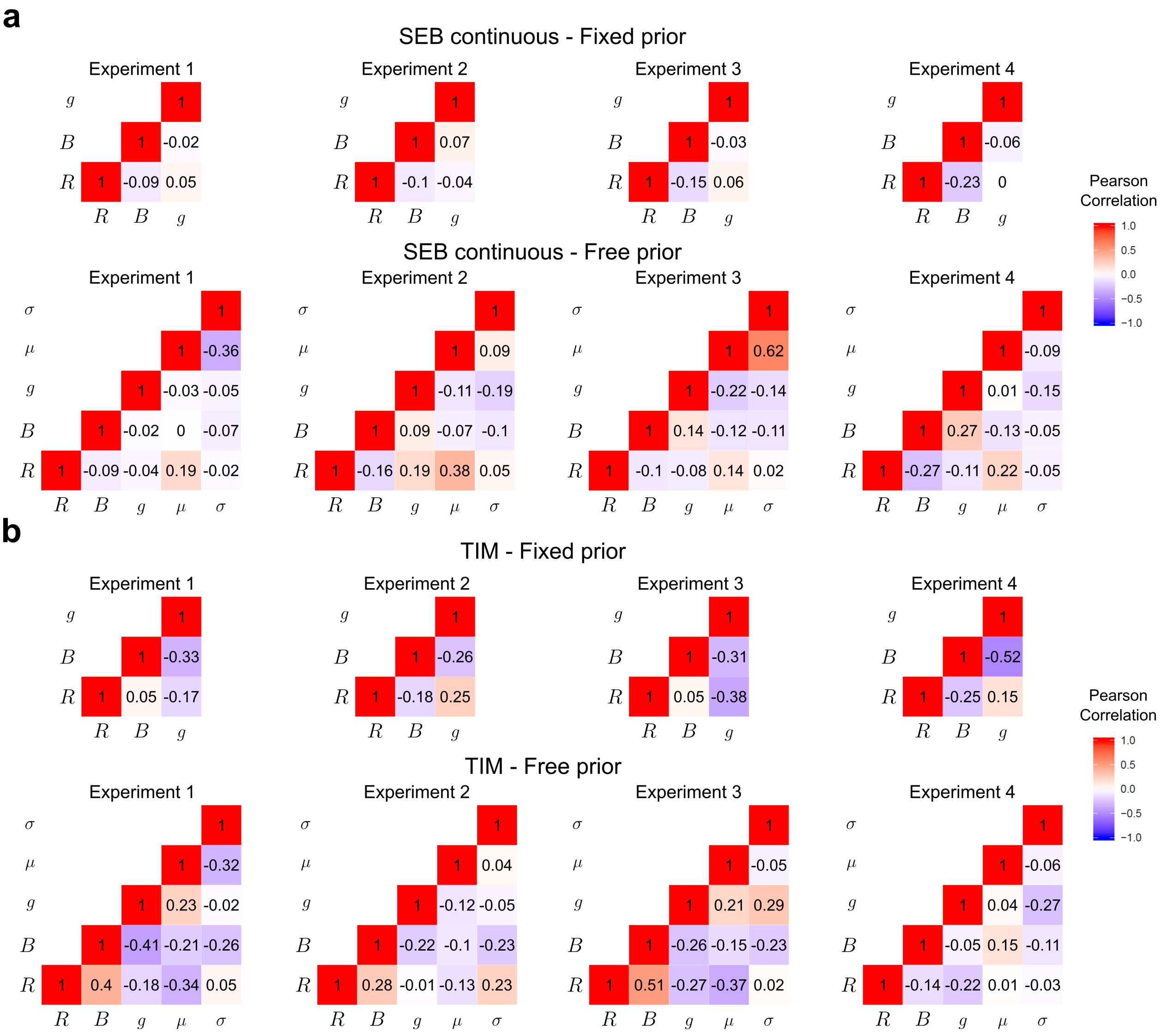

Correlations of parameter fits. **(a)** Pearson correlation of the parameter fits of the SEB continuous for each experiment with fixed (top) and free (bottom) prior parameters. **(b)** Pearson correlation of the parameter fits of the TIM model for each experiment with fixed (top) and free (bottom) prior parameters. The parameters across participants are weekly correlated suggesting that they are identifiable as confirmed in parameter recovery exercises.
