# Supplementary material for: Efficient numerosity estimation under limited time
Source: PLoS Comput Biol. 2025 Mar 7;21(3):e1012790. doi: 10.1371/journal.pcbi.1012790 (PMC12021274; doi:10.1371/journal.pcbi.1012790)
Supplement: S1 Table — (PDF) [file pcbi.1012790.s007.pdf]

Supplementary Table 1

Prior parameters	Model	Experiment	R	B	g	μ	σ
Fixed	TIM	1	35.0	3.03	0.014		
		2	31.7	2.89	0.038		
		3	56.0	3.04	0.014		
		4	28.1	2.75	0.041		
	SEB discrete	1	13.4	7.09	0.010		
		2	10.6	8.68	0.012		
		3	13.7	7.62	0.014		
		4	8.5	6.71	0.029		
	SEB continuous	1	13.4	6.00	0.006		
		2	10.3	4.98	0.002		
		3	14.0	5.47	0.002		
		4	8.4	5.17	0.014		
Free	TIM	1	21.9	1.89	0.009	0.78	1.38
		2	20.1	1.73	0.010	1.35	1.74
		3	27.0	1.96	0.010	-1.17	2.51
		4	11.5	1.72	0.013	1.01	2.33
	SEB discrete	1	11.4	9.26	0.010	1.53	0.93
		2	10.7	8.04	0.009	1.43	0.99
		3	13.8	7.81	0.025	1.34	0.98
		4	7.7	9.24	0.013	1.00	1.19
	SEB continuous	1	13.9	6.18	0.010	1.29	0.64
		2	12.9	6.33	0.005	1.30	0.68
		3	14.7	5.52	0.004	1.42	0.77
		4	10.3	5.08	0.019	0.84	1.22

Parameter fits. Highest density of each parameter at the population level for each model and experiment.
