# Supplementary material for: Efficient numerosity estimation under limited time
Source: PLoS Comput Biol. 2025 Mar 7;21(3):e1012790. doi: 10.1371/journal.pcbi.1012790 (PMC12021274; doi:10.1371/journal.pcbi.1012790)
Supplement: S2 Table — (PDF) [file pcbi.1012790.s008.pdf]

Supplementary Table 2

Prior parameters	Model	Expt. 1	Expt. 2	Expt. 3	Expt. 4	All Expts.
Fixed	TIM	55 136	63 016	56 126	68 453	242 731
	SEB discrete	54 660	59 113	55 318	64 796	233 887
	SEB continuous	**53 663**	**57 732**	**53 810**	**63 347**	**228 552**
Free	TIM	52 665	58 105	53 415	62 582	226 767
	SEB discrete	52 536	55 954	52 427	62 247	223 165
	SEB continuous	**50 978**	**54 420**	**50 486**	**60 444**	**216 328**

AIC of the model fit. The continuous version of SEB has the lowest AIC when the prior parameters are either fixed or free, which indicates a better fit to the behavioral data. In addition, the versions of the models with free prior parameters have lower AICs than the versions with fixed prior parameters. (Expt: Experiment).
